# High Concentrate Supplementation during Late Pregnancy and Lambing Reduced Mortality of Triplet-Bearing Maternal Ewes

**DOI:** 10.3390/ani14162302

**Published:** 2024-08-08

**Authors:** Emmanuelle Haslin, Sarah E. Blumer, Darren Gordon, Gavin A. Kearney, Paul R. Kenyon, Lyndon J. Kubeil, Gordon Refshauge, Jason P. Trompf, Andrew N. Thompson

**Affiliations:** 1 Centre for Animal Production and Health, Murdoch University, 90 South St, Murdoch, WA 6150, Australia; andrew.thompson@murdoch.edu.au (A.N.T.); 2 Nextgen Agri Ltd., 61 Ngaio Street, Saint Martins, Christchurch 8022, New Zealand; 3 36 Paynes Road, Hamilton, VIC 3300, Australia; 4 School of Agriculture and Environment, Massey University, Tennent Drive, Palmerston North 4410, New Zealand; p.r.kenyon@massey.ac.nz; 5 Agriculture Victoria, 89 Sydney Road, Benalla, VIC 3672, Australia; 6 New South Wales Department of Primary Industries, 296 Binni Creek Road, Cowra, NSW 2351, Australia; gordon.refshauge@dpi.nsw.gov.au; 7 Agri-Source, 2A Bradley Drive, Melbourne, VIC 3082, Australia

**Keywords:** triplet-born lambs, ewe mortality, lamb survival, feed-on-offer, supplementary feeding, paddock topography

## Abstract

**Simple Summary:**

This study investigated the effects of feed-on-offer (FOO) and supplementation with concentrates during late pregnancy and lambing on the survival of triplet-bearing ewes and their lambs in Australia. High and low levels of FOO and concentrate supplementation during late pregnancy and lambing were tested on 10 commercial farms between 2019 and 2021 using 1772 triplet-bearing Maternal ewes. Lamb survival and ewe mortality were estimated at lamb marking. Survival of triplet-born lambs was not impacted by levels of FOO or supplementation. Triplet-bearing ewes receiving high levels of supplementation had a 40% decrease in mortality to marking compared with those receiving lower levels of supplementation. These findings suggest no additional benefits to survival of triplet-bearing ewes when FOO levels exceed 1200 kg DM/ha during late pregnancy and lambing, but increased supplementation with concentrates can reduce ewe mortality.

**Abstract:**

Low survival of triplet-bearing ewes and their lambs represents lost production and a welfare issue. The effects of feed-on-offer (FOO; low: 1205 vs. high: 1980 kg DM/ha) and concentrate supplementation (low: 50 vs. high: 300+ g/ewe/day) levels during late pregnancy and lambing on the survival of triplet-bearing ewes and their lambs were investigated on 10 commercial farms using 1772 triplet-bearing Maternal ewes. Ewe and lamb survival were estimated at marking, and ewe body condition score (BCS) was recorded in late pregnancy and at marking. Although FOO treatment had no effect on triplet-bearing ewe mortality, receiving higher supplementation decreased mortality by 40% and increased BCS at marking by 0.14 compared with a lower supplementation (*p* < 0.05). Supplementation, FOO treatments, weather conditions during lambing and shelter availability had no effect on triplet-lamb survival. These findings suggest no additional benefit to triplet-bearing ewe survival when FOO levels exceed 1200 kg DM/ha during late pregnancy and lambing, but increased supplementation can reduce ewe mortality. Further research is required to determine the response to the supplementation level at lower FOO levels on triplet-bearing Merino ewes and their lambs and establish whether supplementation of triplet-bearing ewes during late pregnancy and lambing with higher levels of concentrates would be cost-effective.

## 1. Introduction

Increasing fecundity can increase farm profitability, but it also results in a greater proportion of ewes bearing triplet lambs within a flock [1,2]. Triplet lambs are lighter at birth, are offered less milk and colostrum, and can be metabolically challenged compared to twin lambs, leading to a greater mortality to weaning [3,4]. Ewe mortality is also greater in triplet-bearing ewes during late pregnancy and early lactation than single- and twin-bearing ewes [3,5]. Survey data of Australian commercial farms indicated the triplet-bearing ewe mortality between pregnancy scanning and early lactation averaged 6.4%, while the survival rate of triplet lambs was 59% [5]. A contributor to this high ewe mortality is likely to be the gap between the ewe feed intake and the high nutritional demand during late pregnancy and early lactation [3]. Due to the significant growth and development of the combined foetuses in late pregnancy, triplet-bearing ewes have limited rumen space compared to ewes carrying fewer foetuses [6]. This can limit their feed intake during late pregnancy, and mobilisation of maternal energy reserves is inevitable, especially where paddock feed is limited in quantity and or quality.

Optimal grazing guidelines for single-, twin- and triplet-bearing Maternal ewes during pregnancy and lactation have been well established for extensive grazing management conditions in New Zealand [7,8,9,10]. While in Australia, the body condition score (BCS) targets are reported for single- and twin-bearing Maternal ewes during pregnancy and lactation as an indicator of the level of required nutrition [11]. To our knowledge, however, there is no information about the levels of feed-on-offer (FOO) required during late pregnancy and lambing in Australia to minimise mortality of triplet-bearing ewes and their lambs. The use of supplements in addition to unrestricted grazing conditions in late pregnancy and lactation of triplet-bearing ewes has been examined [12,13,14]. In these studies, no effect on triplet-lamb survival was observed, however, the number of animals was relatively low. The impact of using concentrate supplements on triplet-bearing ewe mortality was not studied, nor were the responses to concentrate supplementation when available pasture was limited.

In a recent survey, sheep producers across southern Australia identified the level of FOO during lambing as a priority for research to increase the survival of triplet-bearing ewes and their lambs [5]. The survey results suggested that FOO targets for triplet-bearing ewes during lambing ranged from 800 to 2500 kg dry matter (DM)/ha, reflecting uncertainty and the absence of specific guidelines for triplet-bearing ewes, and/or the significant variation in environmental conditions between late pregnancy and lambing across southern Australia [5]. The present study was therefore designed to investigate the combined effects of feeding concentrate supplements during late pregnancy and FOO between late pregnancy and early lactation (i.e., lamb marking) on the survival of triplet-bearing ewes and their lambs under Australian conditions. It was hypothesised that survival of triplet-bearing ewes and lambs would be improved when (i) they were grazing on higher FOO levels during late pregnancy and lambing and (ii) they were fed higher levels of concentrate supplementation during late pregnancy and lambing, irrespective of FOO levels.

## 2. Materials and Methods

### 2.1. Sites and Experimental Design

**Figure 1:**
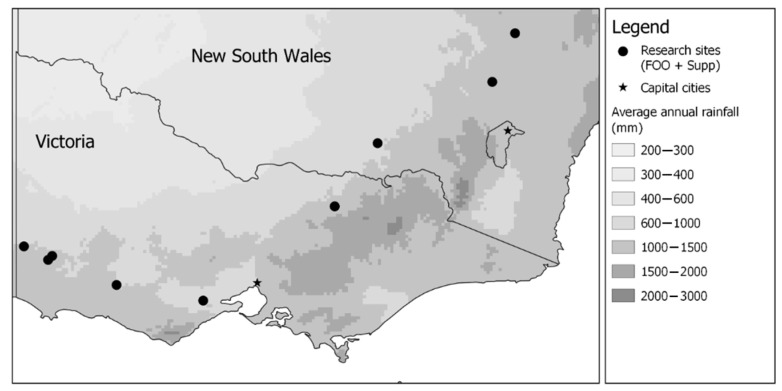
Location of the research sites (10 farms). One site was used in both 2020 and 2021.

**Figure 2:**
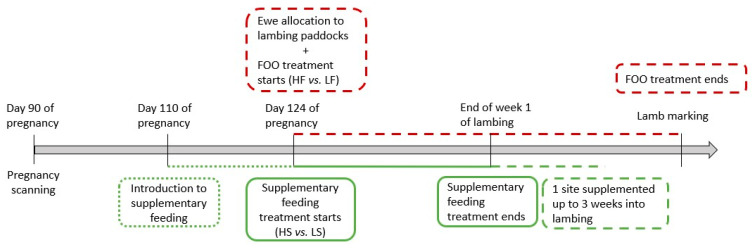
Timeline of the experimental design. FOO: feed-on-offer (kg Dry Matter/ha); HF: high feed-on-offer; LF: low feed-on-offer; HS: high supplementary feeding rate; LS: low supplementary feeding rate.

This study was implemented on 10 commercial farms with Maternal ewes across New South Wales (3 farms) and Victoria (7 farms) between 2019 and 2021 (Figure 1). One farm was used in both 2021 and 2020 and was included as separate sites. Maternal refers to composite sheep breeds or crossbreds used for prime lamb production. The study tested a 2 × 2 factorial combination of feed-on-offer (FOO; high (HF) or low (LF)) and supplementation levels (high (HS) or low (LS)). A total of 1772 triplet-bearing Maternal ewes were allocated randomly to the following treatments at 124 days on average from the introduction of the rams (i.e., start of joining; Figure 2): high FOO and high level of supplementation (HFHS), high FOO and low level of supplementation (HFLS), low FOO and high level of supplementation (LFHS) and low FOO and low level of supplementation (LFLS).

Triplet-bearing ewes were identified by transabdominal ultrasound at approximately day 90 of pregnancy and selected for this study at day 110 from the introduction of the rams. On each farm, ewes were then managed as a single mob and introduced to concentrate supplementation until they were randomly allocated to a treatment and lambing paddock (124 days from the start of joining). Ewes remained in these paddocks until lamb marking (approximately 200 days from the start of joining). Lamb marking refers to procedures, such as tail docking, castration, vaccination and ear tagging. The average mob size for triplet-bearing ewes during late pregnancy and lambing was 40 ewes with an average stocking rate of 5.3 ewes/ha (Table 1). Ewes in the HS treatment groups were supplemented with a minimum of 300 g/ewe/day whilst those in the LS treatment groups were supplemented with 50 g/ewe/day. Ewes were supplemented with grain or pellets at all sites. Supplementation ceased at the end of the first week of lambing at all sites but one, which continued for up to three weeks into the lambing period (Figure 2).

Lambing paddocks were managed to achieve a difference of at least 500 kg DM/ha between high and low FOO treatments at allocation to lambing paddocks. Management on each farm aimed to achieve a minimum for paddocks allocated to the high FOO treatment and a maximum for paddocks allocated to the low FOO treatment of 1500 kgDM/ha to avoid overlap between treatments. Paddock selection aimed for the stocking rate between treatments to differ by no more than 1 ewe/ha if stocking rates were less than 5 ewes/ha or by no more than 2 ewes/ha if stocking rates were greater than 5 ewes/ha. Lambing paddocks within the research sites were also selected to be similar between treatments in terms of paddock characteristics including topography and the type and amount of shelter (Table 1).

### 2.2. Animal, Pasture and Paddock Measurements

The BCS of ewes [16,17] at each site was assessed by a single operator at allocation to lambing paddocks in late pregnancy and at lamb marking. Ewes had their udder palpated manually at lamb marking to identify their lactation status (i.e., lactating or not). Ewe mortality was calculated for each group based on the number of ewes present at allocation to lambing paddocks and at lamb marking. Lamb survival was calculated for each group based on the number of foetuses identified at pregnancy scanning and the number of lambs present at lamb marking.

Feed-on-offer (kg DM/ha) was visually assessed at 25 locations in each of the lambing paddocks at allocation in late pregnancy and at lamb marking at each site. Pasture composition and the percentage of legumes were also assessed at these times [18]. The visual FOO assessments were calibrated against ten 0.1 m^2^ quadrat cuts from each lambing paddock. Within each quadrat, pasture was harvested to ground level and samples were then dried at 60 °C for at least 48 h, and then weighed to determine the dry matter content as described by [18].

Visual assessments of the lambing paddock characteristics were recorded by a single operator at each site. Characterisation of topography and shelter availability in the lambing paddocks was as described by Lockwood et al. [15]. Paddock topography was also categorised as flat, gently undulating, undulating or rolling based on the main slope of the paddock.

### 2.3. Weather Measurements during the Lambing Period

Data for daily temperature, windspeed and rainfall between day 145 from the ram introduction and lamb marking were collected via the Australian Gridded Climate Data (AGCD) and Australian Community Climate and Earth-System Simulator (ACESS-G) services from the Bureau of Meteorology for each site. The chill index was calculated daily for each site using the formula described by Nixon-Smith [19] to correspond to the weather information issued to Australian sheep producers by the Bureau of Meteorology. The daily windspeed, measured at a height of 10 m, was converted to a lamb height of 0.4 m as per Thornley and Johnson [20]. The daily temperature was calculated using 75% of the maximum and 25% of the minimum daily temperature, as per Horton et al. [21]. The chill index across all research sites averaged 782 ± 5.67 kJ/m^2^/h, with a range from 745 to 803 kJ/m^2^/h. The daily cold snap index was also calculated for each research site and describes the change in the chill index from the preceding day to the current day [22]. The average cold snap across all research sites was 24.5 ± 1.68 kJ/m^2^/h, with a range of 18.9–36.6 kJ/m^2^/h.

### 2.4. Statistical Analysis

Data were analysed using SAS v9.4 (SAS Institute Inc., Cary, NC, USA). All models were examined with statistical significance of terms and interactions thereof accepted at *p* < 0.05. Feed-on-offer, the proportion of legumes in the lambing paddocks, ewe mortality and lamb survival at the paddock level and ewe BCS were analysed using linear mixed models. All models included FOO treatments (HF vs. LF), supplementation treatments (HS vs. LS) and their two-way interaction as fixed effects, and year and site, nested within year, as random effects, resulting in the following equation:

Y = Intercept + β1FOO + β2Supplement + β3(FOO × Supplement) + (1∣Year) + (1∣Year:Site) + ϵ(1)

where β1 is the fixed effect coefficient of FOO treatments, β2 is the fixed effect coefficient of supplement treatments, β3 is the fixed effect coefficient of their two-way interaction and ϵ is the residual error term. The models constructed for ewe mortality and lamb survival at the paddock level also included paddock topography (flat vs. gently undulating vs. undulating vs. rolling) as a fixed effect, the average chill index, average cold snap and total shelter availability as covariates. Paddock characteristics that were not significant (*p* > 0.05) were removed from the final models. The model constructed for ewe BCS at marking included lactation status at lamb marking (lactating vs. not lactating) and the three-way interaction between FOO levels, supplementation levels and lactation status as fixed effects. The three-way interaction was removed from the final model if non-significant (*p* > 0.05).

The probability of a ewe lactating at lamb marking was analysed by fitting a Generalized Linear Mixed Model using a binomial distribution and a logit transformation. The supplementation levels, FOO levels and their two-way interaction were fitted as fixed effects, and year, paddock nested within site and site nested within year were included as random effects.

## 3. Results

### 3.1. Pasture Characteristics

The HF treatment had greater (*p* < 0.01) FOO in late pregnancy and at lamb marking than the LF treatment (Table 2). The proportion of legumes in the pasture was greater in the LF treatment in late pregnancy compared to the HF treatment but did not differ (*p* > 0.05) at lamb marking (Table 2). The FOO and the proportion of legumes in the pasture did not differ (*p* > 0.05) between supplementation treatments or in the two-way interaction between FOO and supplementation treatment (Table 2).

### 3.2. Ewe Mortality to Marking and Lactation Status at Marking

There were no effects of the FOO or supplementation treatments on the proportion of ewes lactating at lamb marking (*p* > 0.05; Table 3). There was no effect of the FOO or the two-way interaction between FOO and the supplementation treatment on triplet-bearing ewe mortality to lamb marking (*p* > 0.05; Table 3). Ewes in the HS treatment groups in late pregnancy had a lower mortality (*p* < 0.05) than ewes in the LS treatment groups, irrespective of FOO (Table 3). The average chill index, cold snap, total shelter available and topography of the lambing paddock had no effect on ewe mortality to lamb marking (*p* > 0.05; data not shown).

### 3.3. Ewe Body Condition Score

The BCS of ewes in late pregnancy did not differ between FOO treatments, supplementation treatments or their two-way interaction (*p* > 0.05; Table 3). At lamb marking, ewes in the LFHS treatment group had a higher BCS than those in the LFLS treatment group (*p* < 0.05; Table 3). Ewes in the HS treatment group had a higher BCS at lamb marking than those in the LS treatment group, irrespective of FOO (Table 3). Ewes that were lactating had a lower BCS at lamb marking than ewes that were not (*p* < 0.001; 2.81 ± 0.08 vs. 3.70 ± 0.09 BCS units, respectively). The three-way interaction between FOO, supplementation treatments and lactation status had no effect on ewe BCS at lamb marking (*p* > 0.05; data not shown).

### 3.4. Lamb Survival

There were no effects of the FOO and supplementation treatments or their two-way interaction on the survival of triplet-born lambs to lamb marking (*p* > 0.05; Table 3). The average chill index, cold snap and total proportion of shelter available in the lambing paddock had no effect on lamb survival (*p* > 0.05; data not shown). Triplet-lamb survival was lower in lambing paddocks with rolling topography than those with gently undulating topography (*p* < 0.05; % (95% confidence interval); 49.5% (35.9–63.2) vs. 69.1% (61.5–76.7), respectively). Lamb survival did not differ between the other topographies (*p* > 0.05; data not shown).

## 4. Discussion

Triplet-bearing ewes supplemented with 300 g/ewe/day or more of concentrates during late pregnancy and lambing had a 40% decrease in mortality compared to ewes supplemented with 50 g/ewe/day, irrespective of the FOO treatment. This partially supported our hypothesis. Furthermore, the BCS at lamb marking was greater (0.14 BCS units) for ewes receiving a high supplementation than those receiving a low supplementation. An increase in BCS in mid- to late pregnancy was reported to decrease the risk of mortality in triplet-bearing ewes [23]. Previous studies reported that supplementing triplet-bearing ewes with 400 g/ewe/day of concentrates in pregnancy increased live weight by 2 kg near parturition, however, no further difference in live weight was observed [12,13]. Kerslake et al. [13] also reported that triplet-bearing ewes that were supplemented had lower plasma beta-hydroxybutyrate and non-esterified fatty acids, indicating that they were under less metabolic stress near parturition than ewes that were not supplemented. Hypocalcaemia and pregnancy toxaemia are the most common metabolic causes of death of ewes during late pregnancy and lambing, irrespective of litter size [24]. Less metabolic stress in late pregnancy, therefore, is likely to contribute to lower triplet-bearing ewe mortality. The high level of supplementation could, therefore, improve triplet-bearing ewe BCS and Maternal ewe survival. More research would be needed to investigate the response of triplet-bearing Merino ewes to a high rate of supplementation during late pregnancy and lactation in terms of their own survival and that of their lambs. Determining an optimum BCS target for triplet-bearing ewes in late pregnancy alongside an economic analysis is required. This analysis would establish whether supplementing triplet-bearing ewes with a higher rate of concentrates in late pregnancy is cost-effective.

The level of FOO during late pregnancy and lambing had no impact on the survival of triplet-bearing ewes when the difference in FOO levels was 800 kg DM/ha. Previous studies in Australia have considered the level of FOO as a potential risk factor for ewe mortality [24,25]. The risk of periparturient traumas was reduced when non-Merino ewes were grazing on 2000 kg DM/ha or more during lambing [24], however, this study did not account for litter size. In the present study, both high and low FOO levels were measured at an average of 1200 kg DM/ha or greater in late pregnancy and at lamb marking, likely explaining the absence of an effect of FOO levels on ewe mortality. Feed intake was not restricted when twin- and triplet-bearing Maternal ewes were offered 1200 kg DM/ha of a ryegrass–white clover sward [10]. This finding is consistent with the absence of a difference in ewe BCS at lamb marking between the high and low FOO levels in the present study. A FOO level of 1200 kg DM/ha in late pregnancy therefore seems to be adequate for triplet-bearing ewes during late pregnancy and lambing and matches the New Zealand recommendations [10].

Feed-on-offer and the level of supplementation with concentrates during late pregnancy and lambing did not impact the survival of triplet-born lambs from Maternal ewes, which did not support our hypothesis. In the present study, triplet-bearing ewes with both high and low FOO levels were provided with unrestricted grazing conditions (1200 kg DM/ha or greater) during late pregnancy and lambing [10], likely explaining the absence of an effect of FOO on lamb survival. Further studies have reported that concentrate supplementation of Maternal ewes in late pregnancy under unrestricted grazing conditions also had no impact on triplet-lamb survival [12,13], which additionally supports the results of the present study. In these studies, twin- and triplet-bearing ewes were either offered 400 g/ewe/day of concentrate sheep pellets from approximately day 100 of pregnancy to parturition or were not offered supplementation. Other studies, however, reported that supplementation with cottonseed or soyabean meal increased the survival of triplet-born lambs [26,27]. Previous studies suggest that due to the multifactorial causes of triplet lamb mortality, only managing the nutrition and condition of triplet-bearing ewes to improve lamb survival would be challenging [3,23]. Other management factors, however, have been reported to improve triplet-lamb survival, such as mob size [28], managing triplet-bearing ewes separately from twin-bearing ewes during lambing [29] and access to shelter [30]. Further research is required to provide robust recommendations on the level of FOO and supplementation during late pregnancy and lactation, combined with the other management factors, including paddock characteristics, to maximise triplet-lamb survival in both the Maternal and Merino breeds.

The chill index during the lambing period did not impact triplet-lamb survival to lamb marking. The chill index in the present study did not present high values on average, which was supported by low cold snap values, indicating relatively mild weather. A high chill index (i.e., ≥960 kJ/m^2^/h [21]) in the first three days of life was previously associated with increased neonatal lamb mortality for single and multiple lambs [21,31]. Other studies, however, reported that lamb survival was not associated with the chill index calculated for the first four weeks of lambing [23,32], which supported our results. The relatively mild conditions in this study in addition to the average of four weeks of lambing could explain the absence of an impact on lamb survival.

Interestingly, a rolling topography of lambing paddocks negatively impacted triplet-lamb survival compared to a gently undulating topography. These topographies were defined by Lockwood et al. [15] as moderate inclines with an approximately 10 to 20° slope for rolling topography and very gentle inclines with an approximately 1 to 5° slope for gently undulating topography. In a rolling topography, the risk of separation of a triplet lamb from its dam may be greater than in a gently undulating paddock, and this might explain the increase in mortality. These results, however, contrasted with previous studies which reported either no effect of paddock topography on the survival of twin- and triplet-born lambs [23,28] or a decrease in lamb survival in paddocks with a 30° slope or greater [33]. The results of the present study must be interpreted with caution, as only two lambing paddocks were classified as rolling. More research would be needed to determine the optimal topography of lambing paddocks for triplet-bearing ewes and their lambs.

## 5. Conclusions

High supplementation during late pregnancy and lambing improved triplet-bearing Maternal ewe survival to lamb marking by 40% and BCS at lamb marking. The survival of triplet lambs, however, was not impacted by the level of FOO, the level of supplementation or their combination during late pregnancy and lambing. Producers should, therefore, provide at least 300 g/ewe/day of concentrate supplementation to triplet-bearing Maternal ewes in late pregnancy and lambing to improve ewe BCS at lamb marking and reduce ewe mortality. Further investigations are required to determine the response to the level of supplementation at different FOO levels of triplet-bearing Merino ewes and their lambs and establish whether feeding a relatively higher rate of concentrate feed to triplet-bearing ewes in late pregnancy would be cost-effective.

## Figures and Tables

**Table 1:** Total number of ewes, average (range) mob size and stocking rate of ewes during the lambing period (ewe/ha), shelter availability within the lambing paddocks (%) for triplet-bearing ewes with the levels of feed-on-offer (FOO; high (HF) or low (LF)) and supplementation (high (HS) or low (LS)) and their interaction ^1^ (treatments at 10 commercial research sites in Australia between 2019 and 2021).

Treatment	Total Ewes	Mob Size	Stocking Rate	Shelter Availability ^2^
HF	894	40.6 (26–63)	5.4 (2.9–8.8)	5.9 (0–30)
LF	878	39.9 (26–63)	5.4 (2.5–11.2)	7.4 (0–30)
HS	873	39.7 (26–53)	5.6 (2.9–11.2)	6.6 (0–30)
LS	899	40.9 (26–63)	5.3 (2.5–7.6)	6.6 (0–30)
HFHS	442	40.2 (26–53)	5.5 (2.9–8.8)	5.8 (0–30)
HFLS	452	41.1 (26–63)	5.3 (2.9–7.6)	6.0 (0–30)
LFHS	431	39.2 (26–53)	5.7 (2.9–11.2)	7.4 (0–30)
LFLS	447	40.6 (28–63)	5.2 (2.5–7.3)	7.3 (0–30)

^1^ HFHS: high FOO/high supplementation, HFLS: high FOO/low supplementation, LFHS: low FOO/high supplementation, LFLS: low FOO/low supplementation. ^2^ The proportion of the lambing paddock occupied by shelter was assessed visually as per Lockwood et al. [15].

**Table 2:** Effects of feed-on-offer (FOO; high (HF) or low (LF)), supplementation (high (HS) or low (LS)) and their interaction ^1^ on FOO and the proportion of legumes in the lambing paddocks in late pregnancy ^2^ and at lamb marking for triplet-bearing ewes at 10 commercial research sites in Australia between 2019 and 2021. Least square means (95% confidence interval).

Treatment	FOO (kg DM/ha)		Legume (%)	
	Late Pregnancy	Lamb Marking	Late Pregnancy	Lamb Marking
HF	1979 (1781–2177) ^b^	2282 (1874–2690) ^b^	19.9 (7.62–32.3) ^a^	22.9 (13.4–32.3)
LF	1205 (1007–1403) ^a^	1328 (920.0–1735) ^a^	27.4 (15.0–39.7) ^b^	27.6 (18.2–37.0)
*p*-value	<0.001	<0.001	0.023	0.123
HS	1591 (1393–1789)	1831 (1424–2239)	23.0 (10.7–35.4)	24.4 (15.0–33.9)
LS	1592 (1394–1790)	1779 (1371–2186)	24.3 (11.9–36.6)	26.0 (16.6–35.4)
*p*-value	0.993	0.560	0.695	0.608
HFHS	1982 (1769–2194)	2245 (1818–2673)	17.7 (4.60–30.8)	21.5 (11.1–31.8)
HFLS	1976 (1764–2188)	2319 (1891–2747)	22.2 (9.06–35.3)	24.3 (13.9–34.7)
LFHS	1202 (989.6–1414)	1417 (989.7–1845)	28.4 (15.2–41.5)	27.5 (17.1–37.8)
LFLS	1208 (995.9–1420)	1238 (810.6–1666)	26.4 (13.2–39.5)	27.7 (17.4–38.1)
*p*-value ^3^	0.993	0.167	0.307	0.673

^a,b^ Means between rows with differing superscripts are different (*p* < 0.05); ^1^ HFHS: high FOO/high supplementation; HFLS: high FOO/low supplementation; LFHS: low FOO/high supplementation; LFLS: low FOO/low supplementation; ^2^ 124 days of pregnancy; ^3^ *p*-value is for the interaction between FOO and supplementation treatments.

**Table 3:** Effects of the feed-on-offer treatment (FOO; high (HF) or low (LF)), supplementation treatment (high (HS) or low (LS)) and their interaction ^1^ on ewe mortality, the proportion of triplet-bearing ewes not lactating at lamb marking, ewe body condition score (BCS) in late pregnancy ^2^ and at lamb marking and the survival of triplet-born lambs at 10 commercial research sites in Australia between 2019 and 2021. Least square means (95% confidence interval).

Treatment	Ewe Mortality (%)	Ewes Not Lactating at Lamb Marking (%)	Ewe BCS		Lamb Survival (%)
			Late Pregnancy	Lamb Marking	
HF	5.86 (3.17–9.30)	6.45 (4.41–9.33)	3.20 (2.95–3.45)	3.25 (3.09–3.41)	60.6 (53.8–67.4)
LF	6.39 (3.57–9.95)	7.46 (5.18–10.6)	3.21 (2.96–3.46)	3.25 (3.09–3.41)	60.6 (53.6–67.6)
*p*-value	0.688	0.414	0.422	0.971	0.984
HS	4.67 (2.30–7.81) ^a^	6.30 (4.30–9.14)	3.21 (2.96–3.46)	3.32 (3.16–3.48) ^b^	60.3 (53.5–67.1)
LS	7.74 (4.61–11.6) ^b^	7.64 (5.32–10.9)	3.20 (2.95–3.45)	3.18 (3.02–3.34) ^a^	60.8 (53.9–67.7)
*p*-value	0.025	0.278	0.507	<0.001	0.788
HFHS	4.90 (2.14–8.71)	6.04 (3.77–9.54)	3.21 (2.95–3.46)	3.28 (3.11–3.45) ^a,b^	59.3 (52.0–66.7)
HFLS	6.89 (3.53–11.3)	6.89 (4.40–10.6)	3.19 (2.94–3.44)	3.22 (3.05–3.39) ^a,b^	61.8 (54.6–69.0)
LFHS	4.44 (1.84–8.12)	6.57 (4.15–10.2)	3.22 (2.97–3.47)	3.36 (3.19–3.53) ^b^	61.3 (54.0–68.6)
LFLS	8.65 (4.85–13.4)	8.46 (5.57–12.7)	3.21 (2.96–3.46)	3.14 (2.97–3.31) ^a^	59.9 (52.2–67.6)
*p*-value ^3^	0.431	0.729	0.902	0.010	0.309

^a,b^ Means between rows with differing superscripts are different (*p* < 0.05); ^1^ HFHS: high FOO/high supplementation; HFLS: high FOO/low supplementation; LFHS: low FOO/high supplementation; LFLS: low FOO/low supplementation; ^2^ 124 days of pregnancy; ^3^ *p*-value of the interaction between FOO and supplementation treatments.

## Data Availability

The datasets generated and/or analysed during the present study are not publicly available but are available from the corresponding author on reasonable request pending permission from the funding body (Meat and Livestock Australia) and Murdoch University.
